# Role of the Polyol Pathway in Locomotor Recovery and Wallerian Degeneration after Spinal Cord Contusion Injury

**DOI:** 10.1089/neur.2021.0018

**Published:** 2021-09-14

**Authors:** Richard J. Zeman, Xialing Wen, Nengtai Ouyang, Abraham M. Brown, Joseph D. Etlinger

**Affiliations:** ^1^ Department of Cell Biology and Anatomy, New York Medical College, Valhalla, New York, USA.; ^2^ MotoGen Inc., Mount Kisco, New York, USA.

**Keywords:** glucose uptake, glucotoxicity, hyperglucosis, locomotor function, polyol pathway, spinal cord injury, Wallerian degeneration

## Abstract

Spinal cord contusion injury leads to Wallerian degeneration of axonal tracts, resulting in irreversible paralysis. Contusion injury causes perfusion loss by thrombosis and vasospasm, resulting in spinal cord ischemia. In several tissues, including heart and brain, ischemia activates polyol pathway enzymes—aldose reductase (AR) and sorbitol dehydrogenase (SDH)—that convert glucose to sorbitol and fructose in reactions, causing oxidative stress and tissue loss. We sought to determine whether activation of this pathway, which has been termed glucotoxicity, contributes to tissue loss after spinal cord contusion injury. We tested individual treatments with AR inhibitors (sorbinil or ARI-809), SDH inhibitor (CP-470711), superoxide dismutase mimetic (tempol), or combined sorbinil and tempol. Each treatment significantly increased locomotor recovery and reduced loss of spinal cord tissue in a standard model of spinal cord contusion in rats. Tissue levels of sorbitol and axonal AR (AKR1B10) expression were increased after contusion injury, consistent with activation of the polyol pathway. Sorbinil treatment inhibited the above changes and also decreased axonal swelling and loss, characteristic of Wallerian degeneration. Treatment with tempol induced recovery of locomotor function that was similar in magnitude, but non-additive to sorbinil, suggesting a shared mechanism of action by reactive oxygen species (ROS). Exogenous induction of hyperglycemia further increased injury-induced axonal swelling, consistent with glucotoxicity. Unexpectedly, contusion increased spinal cord levels of glucose, the primary polyol pathway substrate. These results support roles for spinal glucose elevation and tissue glucotoxicity by the polyol pathway after spinal cord contusion injury that results in ROS-mediated axonal degeneration.

## Introduction

Spinal cord injury (SCI) often leads to Wallerian degeneration and loss of descending and ascending axonal tracts, resulting in irreversible paralysis.^1–3^ These injuries cause hemorrhage and loss of spinal cord perfusion attributable to causes such as thrombosis and vasospasm,^4^ rapidly leading to spinal cord ischemia. In several tissues, including brain and heart, ischemia can activate aldose reductase (AR). AR is the rate-limiting enzyme of the polyol pathway that, together with sorbitol dehydrogenase (SDH), sequentially converts glucose to sorbitol and fructose. These enzymatic reactions promote generation of reactive oxygen species (ROS) and reduce oxidative protection, which cause oxidative stress and consequent tissue loss.^5,6^ AR and SDH are present in both peripheral nerve and spinal cord.^7,8^ Polyol pathway activation is known to cause neuropathy of the peripheral nervous system (PNS), notably in diabetes with loss of axons,^9^ which suggests that similar deleterious effects may also occur in the spinal cord.

Wallerian degeneration involves the formation of foci of axonal swelling that is more rapid and prominent in the central nervous system (CNS) compared to the PNS,^10^ leading to axonal dysfunction, discontinuity, and fragmentation. A hallmark of polyol pathway activity in the PNS is axonal swelling and degeneration as observed in diabetic neuropathy.^11–13^ The mechanisms involved in Wallerian degeneration have only been partially elucidated, but there is evidence for involvement of oxidative stress given that it can be opposed by antioxidants.^14^

Given that prominent axonal swelling and degeneration leading to axonal loss were observed in diabetic neuropathy**,** we hypothesized that a similar mechanism of neurodegeneration may underlie the Wallerian degeneration of myelinated axonal tracts after spinal cord contusion injury. In diabetic neuropathy, evidence from knockout mice or pharmacological inhibitors indicates that activation of AR, which converts glucose to sorbitol in a process that causes oxidative stress, is necessary for subsequent axonal loss.^9^ We postulated that SCI might also activate AR, causing axonal degeneration and loss, given that the enzyme can be activated by ischemia attributable to injury.^5,6^

The purpose of this study was to determine whether activation of the glucose/polyol pathway occurs in the spinal cord after contusion injury, the most common type of spinal injury, in a standard experimental animal model of spinal cord contusion injury. A second goal was to test whether inhibition of the polyol pathway can improve recovery of locomotor function and spare spinal cord tissue after contusion. Our studies found evidence of both elevated spinal cord glucose, which we term hyperglucosis, and polyol pathway activation after contusion injury, resulting in elevated levels of polyols and ROS. Inhibition of glucotoxicity with polyol pathway blockers improved locomotor recovery and opposed Wallerian degeneration, suggesting that these agents are a potential therapeutic modality for SCI.

## Methods

The Institutional Animal Care and Use Committee of New York Medical College (Valhalla, NY) approved all of the procedures involving vertebrate animals. Adult female (∼240 g) Wistar rats were obtained from Charles River Breeding Laboratories (Wilmington, MA) and housed in a temperature-regulated (23°C) animal facility. As in our previous studies,^15–17^ spinal cords were contused with a weight drop apparatus at the level of T10. Before surgery, rats were anesthetized with pentobarbital sodium (40 mg/kg, intraperitoneal [i.p.]), and the completeness of anesthesia was verified by toe foot and tail pinch. Laminectomy was then performed aseptically at T9–T10 to expose the spinal cord. Spinous processes at T8 and T11 were fixed with clamps to prevent movement during contusion. Body temperature was maintained at 37°C with a temperature-controlled heating pad and rectal thermometer. Contusion injuries were produced in rats by dropping a guided 10-g rod with a tip diameter of 2.5 mm, as described for the NYU impactor,^18^ from a height of 25 mm onto the exposed dura.

After contusion, each incision was closed with surgical staples. Bladders were expressed manually twice-daily for several weeks until automaticity returned. Six groups of contused rats (*n* = 9–12) were subsequently treated with either ARI-809 (50 mg/kg orally in 1 mL of H_2_O at 20 min and 24 and 48 h post-injury; Pfizer, Groton, CT), sorbinil (65 mg/kg orally in 1 mL of H_2_O at 20 min and 24 and 48 h post-injury; Pfizer), CP-470711 (5 mg/kg orally in 1 mL of H_2_O at 20 min and 24 and 48 h post-injury; Pfizer), tempol (275 mg/kg i.p. at 20 min post-injury; Sigma-Aldrich, St. Louis, MO), or sorbinil and tempol in combination or were untreated. Hyperglycemia was induced in another group of contused rats by administration of oral glucose (2 g/kg/h) at −0.5, 1, and 2 h post-injury.

### Behavioral analysis

Recovery of locomotor function was determined with the Basso, Beattie, and Bresnahan (BBB) locomotor scale.^19,20^ For 3 consecutive days after contusion and at 1-week intervals thereafter for up to 6 weeks post-injury, each rat was scored for locomotor function according to the BBB scale. The average score for both hindlimbs of each animal was assigned by two observers without knowledge of the treatment condition during each 4-min session of open field testing.

### Spinal cord histomorphometry

Immediately after the final behavioral evaluation, spinal cords were fixed by transcardial perfusion in anesthetized rats (pentobarbital sodium, 40 mg/kg, i.p.) with phosphate-buffered saline (pH 7.4) containing 4% paraformaldehyde, dissected, and embedded in paraffin for serial sectioning with a microtome. Spinal cords were sectioned transversely from T9 to T11 in 10-mm blocks, including the contusion site, which could be visualized externally. The contusion site was sectioned transversely throughout at a thickness of 15 μm and stained for myelin with luxol fast blue and counterstained with cresyl violet, as previously described.^15,19^ Quantification of the cross-sectional area of spared spinal cord tissue was performed with planimetry software (ImageJ; National Institutes of Health, Bethesda, MD) from digitized images (Photometrics, Tucson, AZ) of the stained sections at 0.5-mm intervals extending 3–4 mm rostrally and caudally from the contusion epicenter, that is, the section exhibiting the largest lesion, from each spinal cord without knowledge of the treatment condition.

Immunohistochemistry was performed with mouse monoclonal antibody to neurofilament 200 (NF-200; 1:100; Sigma-Aldrich) or mouse polyclonal antibody to AR (AKR1B10, 1:100; Novus, Centennial, CO) and visualized with either biotinylated antimouse or -rabbit immunoglobulin G and streptavidin-conjugated horseradish peroxidase (Zymed, San Francisco, CA) in deparaffinized transverse sections at the contusion epicenter. Digitized images of two random fields (120 × 160 μm) within a section of the ventral white matter from each rat were obtained with a 40 × objective and examined in Adobe Photoshop CS (V 8.0; Adobe, San Jose CA) to determine luminance attributable to peroxidase staining with diaminobenzamine (DAB).

After color sampling of DAB-stained structures, color select was used to outline the areas occupied by DAB-stained axons. The luminosity function was then used to determine axonal AR immunoreactivity. Density and cross-sectional areas of NF-200 immunoreactive axons were measured by particle analysis (ImageJ; National Institutes of Health) within each image. Longitudinal sections of ventral white matter at the contusion epicenter were immunostained for NF-200 to examine axons undergoing Wallerian degeneration.

### Glucose and sorbitol assays

Spinal cord segments (1 cm), including the contusion region, were weighed, homogenized in 2 M of ice-cold HClO_4_, and centrifuged (80,000*g*, 10 min) to obtain the supernatants that were neutralized with 2 M of KOH. Aliquots of supernatants were enzymatically assayed for glucose with the hexokinase method,^21^ which generates glucose-6-phosphate and β-nicotinamide adenine dinucleotide 2′-phosphate, reduced (NADPH) through glucose-6-phosphate dehydrogenase activity detected spectrophotometrically at 340 nm (Sigma-Aldrich). Supernatant aliquots were also assayed for sorbitol^22^ by oxidation to fructose in the presence of sorbitol dehydrogenase (SDH) and nicotinamide adenine dinucleotide (NAD^+^) with the formation of reduced NAD^+^ (NADH). In the presence of diaphorase, NADH reduces iodonitrotetrazolium to formazan detected at 492 nm (Megazyme, Chicago, IL). Plasma obtained from blood samples was enzymatically assayed for glucose with the hexokinase method.

### Statistical analysis

Statistical significance of the effects of the treatments on locomotor scoring were determined by mixed factorial analysis of variance with repeated measures and weekly scores by one-way analysis of variance followed by Duncan's test multiple range *post hoc* (IBM-SPSS STATISTICS 27; SPSS, Inc., Chicago IL). Between-group comparisons of cross-sectional areas of spared spinal cord tissue, axon density and areas, AR immunoreactivity, and glucose and sorbitol concentrations were performed with one- or two-way analysis of variance and Duncan's test *post hoc*. Statistical significance was determined at the *p* < 0.05 level.

## Results

**FIG. 1.**
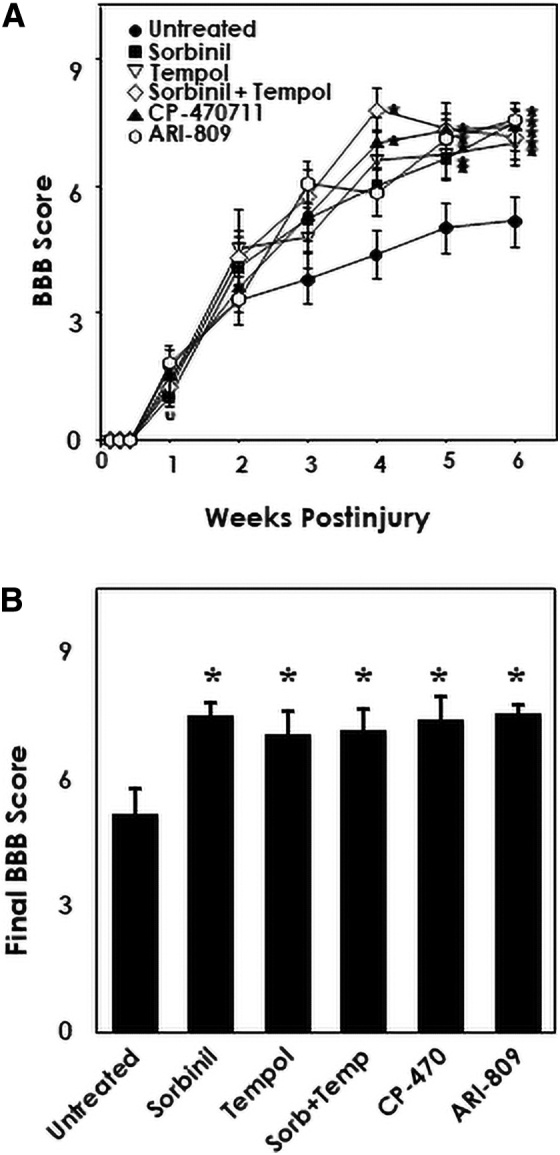
Inhibition of aldose reductase with sorbinil or ARI-809 and sorbitol dehydrogenase with CP-470711 increases locomotor recovery after spinal cord contusion injury similarly to treatment with the antioxidant nitroxide, tempol. (**A**) Values are means ± SE of determinations of locomotor recovery according to the BBB locomotor scale for 6 weeks post-injury. Six groups of rats (*n* = 9–12) received contusion injury with a weight drop apparatus and received ARI-809 (50 mg/kg orally at 20 min and 24 and 48 h post-injury), sorbinil (65 mg/kg orally at 20 min and 24 and 48 h post-injury), CP-470711 (5 mg/kg orally at 20 min and 24 and 48 h post-injury), tempol (275 mg/kg i.p. at 20 min post-injury), or sorbinil and tempol in combination or were untreated as shown. *p < 0.05, significant effects of sorbinil, ARI-809, CP-470711, tempol, or sorbinil and tempol in combination on BBB scores compared to untreated injured rats (*n* = 9–12; mixed factorial analysis of variance with repeated measures and Duncan's test *post hoc*). (**B**) Final BBB scores as in (A). BBB, Basso, Beattie, and Bresnahan; i.p., intraperitoneal; SE, standard error.

To determine whether inhibition of the polyol pathway protects from loss of locomotor function after SCI, groups of rats received spinal cord contusion and were treated with either the AR inhibitors, sorbinil or ARI-809, or the SDH inhibitor, CP-470711. Consistent with our hypothesis, inhibitors of either AR or SDH, enzymes of the polyol pathway, increased recovery of locomotor function according to the BBB locomotor scale to a similar extent compared to untreated injured rats (Fig. 1A,B). Recovery of locomotor function induced by polyol pathway inhibitors was comparable in magnitude to the effect of treatment with the superoxide dismutase (SOD) mimetic, tempol (Fig. 1A,B), that was previously shown to enhance locomotor recovery.^17^ The effect of tempol was also non-additive with sorbinil when used in combination (*p* < 0.05), suggesting a common end-point of reduced ROS.

**FIG. 2.**
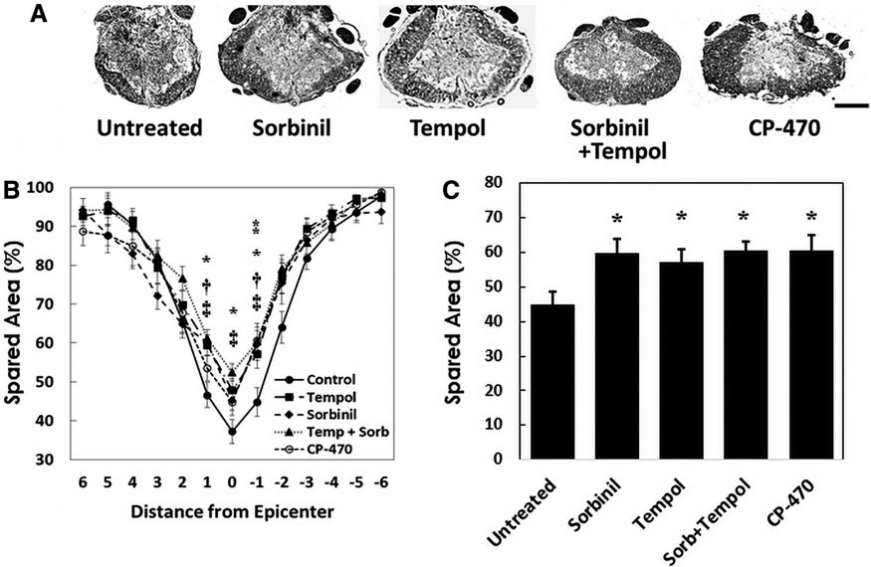
Inhibition of aldose reductase with sorbinil and sorbitol dehydrogenase with CP-470711 spares spinal cord tissue after spinal cord contusion injury similarly to treatment with the antioxidant nitroxide, tempol. (**A**) Transverse sections of spinal cords at the contusion epicenter from sorbinil, CP-470711, tempol, or combined sorbinil and tempol treated or untreated rats at 6 weeks after contusion injury, as shown. Rats received contusion injury and treatment as described for Figure 1. Sections were stained with luxol fast blue for myelin and counterstained with cresyl violet as described in Methods. Typically, contusion injury resulted in a centrally located lesion within the spinal cord with areas of gliosis and cyst formation or cavitation with a peripheral rim of remaining white matter. The central cavity and peripheral rim of spared tissue appeared histologically similar in treated and untreated spinal cords, although a greater extent of sparing of spinal cord white matter was apparent after contusion of treated compared to untreated spinal cords. The calibration bar equals 0.5 mm; *n* = 9–12. (**B**) Values are means of determinations of the cross-sectional area of spared spinal cord tissue expressed as a percentage (%) of total cross-sectional area as a function of distance (mm) rostrally and caudally from the contusion epicenter. Increases in % spared area attributable to ^**†**^sorbinil, ^⁑^CP-470711, *tempol, or combined ^**‡**^sorbinil and tempol treatment (*p* < 0.05, one-way analysis of variance and Duncan's test *post hoc*; *n* = 9–12). (**C**) Spared area (%) at 1 mm caudal to the epicenter. *p < 0.05, significant increases in % spared area attributable to sorbinil, CP-470711, tempol, or combined sorbinil and tempol treatment (one-way analysis of variance and Duncan's test *post hoc*).

Spinal cord contusion injury characteristically results in a centrally located lesion within the spinal cord containing areas of gliosis and cyst formation or cavitation and a peripheral rim of remaining white matter (Fig. 2A). The extent of this sparing of spinal cord tissue has been quantitatively related to recovery of locomotor function, as determined with the BBB scale, consistent with a role of surviving axonal tracts in supporting recovery of locomotor function.^15–17,19,23^ In agreement with these findings, treatment with AR inhibitor sorbinil, SDH inhibitor CP-470711, tempol, or sorbinil and tempol in combination increased sparing of spinal cord tissue to a similar extent (Fig. 2B,C) compared to untreated spinal cords (*p* < 0.05). However, tempol did not further increase sparing of spinal cord tissue when used in combination with sorbinil (*p* > 0.05). Overall, increases in spared spinal cord tissue (28–35%) attributable to treatment with these agents were paralleled by increases (36–46%) in BBB scores.

**FIG. 3.**
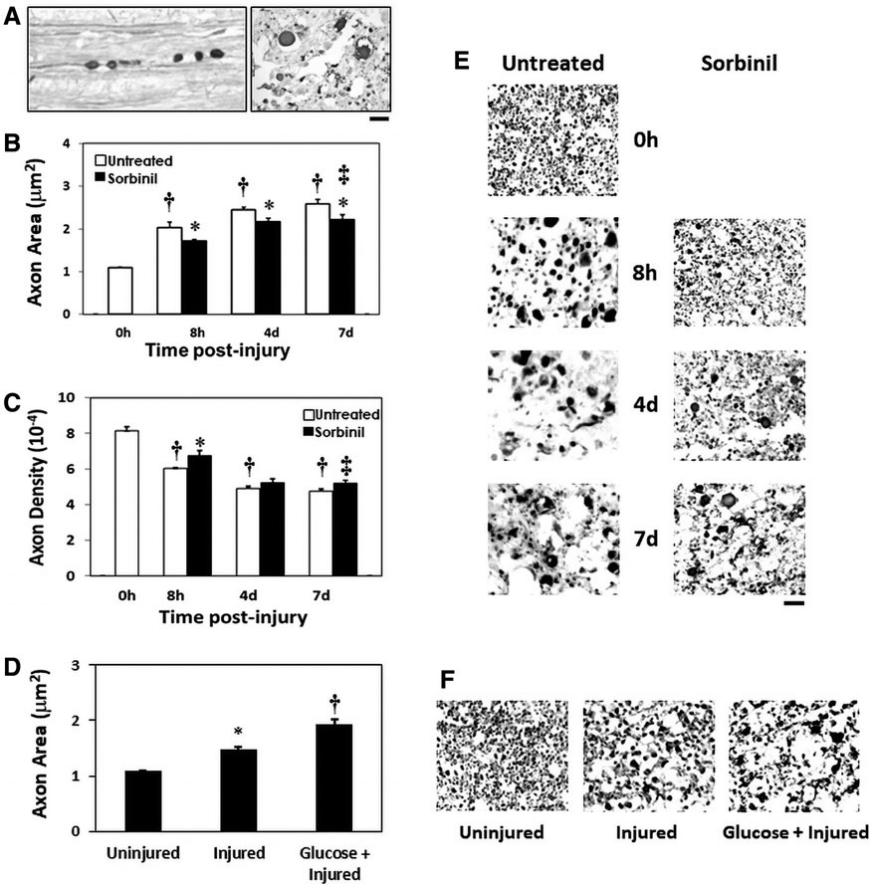
Inhibition of aldose reductase with sorbinil decreases Wallerian degeneration of axons, and hyperglycemia increases axonal swelling at the spinal cord contusion epicenter. (**A**) Left: micrograph of a longitudinal section of ventromedial white matter at the contusion epicenter with NF-200 immunopositive axon exhibiting segmental swelling (“beading”) leading to discontinuity and Wallerian degeneration. Right: micrograph of transverse section of ventromedial white matter at the contusion epicenter with NF-200 immunopositive axons in different stages of swelling and degeneration. Both images were acquired at 7 days post-contusion. The calibration bar equals 10 μm. (**B**) Mean cross-sectional area of axons in the ventromedial white matter at the contusion epicenter. Rats that were treated with sorbinil (65 mg/kg/d) or were untreated received contusion injury with a weight drop apparatus, and the progressive swelling of NF-200 immunopositive axons was determined at 0 (uninjured) and 8 h and 4 and 7 days post-injury. ^†^*p* < 0.05, significant increases in axonal cross-sectional area attributable to contusion injury in untreated rats at 8 h and 4 and 7 days post-injury compared to laminectomized but uninjured rats (0 h; one-way analysis of variance and Duncan's test *post hoc*). ^‡^*p* < 0.01, significant reductions in axonal cross-sectional area at 8 h and 4 and 7 days post-injury by sorbinil treatment (65 mg/kg/d; two-way analysis of variance) and *p < 0.05 (Duncan's test *post hoc*; *n* = 6–8; 0 h, *n* = 3). (**C**) Values are means (±SE) of determinations of axon density in ventromedial white matter at the contusion epicenter as above. ^†^*p* < 0.05, significant decreases in axonal density attributable to contusion injury in untreated rats at 8 h and 4 and 7 days post-injury compared to laminectomized but uninjured rats (0 h; one-way analysis of variance and Duncan's test *post hoc*). ^‡^*p* < 0.01, decreased axonal density attributable to contusion was significantly reduced by sorbinil treatment (65 mg/kg/d; two-way analysis of variance) and *p < 0.05 (Duncan's test *post hoc*). (**D**) Mean cross-sectional area of axon ventromedial white matter at the contusion epicenter determined from NF-200 immunopositive axons as above showing increased axonal swelling at 3 h post-injury. *p < 0.05, significant increase in axonal cross-sectional area attributable to contusion injury in untreated rats at 3 h post-injury compared to laminectomized but uninjured rats (0 h; one-way analysis of variance). ^†^*p* < 0.05, significant increase in axonal cross-sectional area at 3 h post-injury compared to untreated injured rats by oral glucose (2 g/kg/h; one-way analysis of variance; uninjured, *n* = 3; injured, *n* = 3; glucose + injured, *n* = 4). (**E**) Micrographs of transverse sections of contusion epicenter ventromedial white matter NF-200 immunopositive axons in different stages of swelling and degeneration at post-injury times as indicated. Reduced levels of NF-200 immunopositive axons post-injury are observed in sorbinil-treated animals, as shown. The calibration bar equals 10 μm for panels (E) and (**F**). (F) Micrographs of transverse sections of contusion epicenter ventromedial white matter NF-200 immunopositive axons in different stages of swelling and degeneration uninjured and 3 h post-injury, with or without added glucose treatment, as indicated. NF-200 immunopositive axon areas increased post-injury and were further increased by glucose administration as shown. NF-200, neurofilament 200; SE, standard error.

Wallerian degeneration, which contributes to the loss of axonal tracts and paralysis post-SCI, is more rapid and prominent in the CNS compared to the PNS.^10^ This mode of axonal degeneration involves the formation of foci of swelling (“beading”) along axons leading to axonal fragmentation, discontinuity (Fig. 3A), and dysfunction. In order to quantify the time course and extent of Wallerian degeneration post-SCI, measurements of axonal swelling and density were performed in transverse sections of ventromedial white matter immunostained for axonal NF-200 at the contusion site. All determinations were made during the first week after contusion injury, when loss of spinal cord white matter and axonal degeneration primarily occurs.^23^ The ventromedial axonal tracts are relatively spared after spinal cord contusion; nonetheless, injury led to a progressive swelling of axons (Fig. 3B), as indicated by increased axonal cross-sectional area (86–136%) during 1 week post-injury. Spinal injury also led to a progressive (26–41%) loss of axons (Fig. 3C), as indicated by decreased axonal density during this period, consistent with ongoing Wallerian degeneration. Treatment with sorbinil reduced axonal swelling to 57–103% and axon loss to 17–36%.

In order to demonstrate a role of glucose in the axonal swelling characteristic of Wallerian degeneration after contusion injury, hyperglycemia was induced by administration of oral glucose (2 g/kg/h) at −0.5, 1, and 2 h post-injury. Blood glucose levels 3 h post-injury were elevated to 379 ± 46 mg/dL compared to 157 ± 35 mg/dL in injured untreated rats (*p* < 0.05). Measurement of axonal cross-sectional area was performed, as in Figure 3B, in transverse sections of ventromedial white matter immunostained for axonal NF-200 at the contusion site (Fig. 3D). Mean axonal cross-sectional area increased 36% at 3 h post-injury in contused spinal cords compared to uninjured spinal cords. Hyperglycemia increased axonal cross-sectional area an additional 40% at 3 h post-injury compared to untreated injured spinal cords, consistent with promotion of axonal swelling by spinal cord hyperglucosis post-injury.

**FIG. 4.**
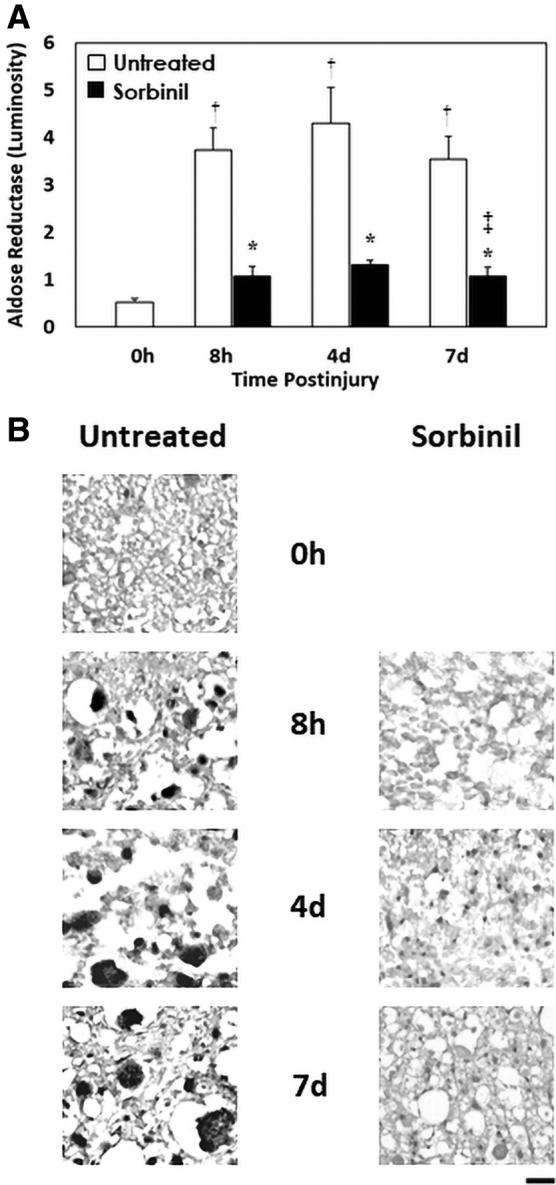
Spinal cord contusion increases axonal aldose reductase (AKR1B10) immunoreactivity at the contusion site, which is mostly prevented by sorbinil. (**A**) Mean values (±SE) of measurements of axonal aldose reductase (AKR1B10) immunoreactivity in ventromedial white matter at the contusion epicenter as described in Methods. ^†^*p* < 0.05, significant increases in axonal aldose reductase immunoreactivity attributable to contusion injury in untreated rats at 8 h and 4 and 7 days post-injury compared to laminectomized but uninjured rats (0 h; one-way analysis of variance and Duncan's test *post hoc*). ^‡^*p* < 0.01, increased axonal aldose reductase (AKR1B10) immunoreactivity attributable to contusion was significantly reduced by sorbinil treatment (65 mg/kg/d; two-way analysis of variance) and *p < 0.05 (Duncan's test *post hoc*; uninjured, *n* = 7; untreated, *n* = 3–4; sorbinil, *n* = 3–7). (**B**) Micrographs of transverse sections of contusion epicenter ventromedial white matter with aldose reductase (AKR1B10) immunopositive axons in different stages of swelling and degeneration at post-injury times as indicated. Reduced levels of aldose reductase positive axons post-injury observed in sorbinil-treated animals, as shown. The calibration bar equals 10 μm. SE, standard error.

The possible involvement of AR in the axonal swelling and degeneration that occurs after contusion injury was examined. Transverse sections of ventromedial white matter at the contusion site were examined for AR immunoreactivity. Axons in different stages of swelling and degeneration were immunopositive for AKR1B10 (Fig. 4B), an AR that is a member of the aldo-keto reductase superfamily. During the first week post-injury, 580–720% increases in axonal AKR1B10 immunoreactivity were observed at 8 h and 4 and 7 days post-injury (Fig. 4A) compared to laminectomized, but uninjured, rats. Sorbinil treatment reduced axonal AKR1B10 immunoreactivity attributable to contusion to only 104–153% versus uninjured.

**FIG. 5.**
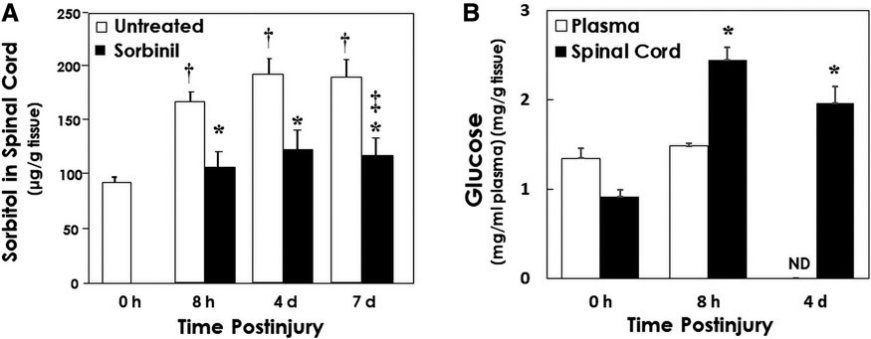
(**A**) Spinal cord contusion increases sorbitol levels at the contusion site, which is mostly prevented by the aldose reductase inhibitor, sorbinil. Mean values (±SE) of measurements of sorbitol concentration in spinal cord segments centered at the contusion epicenter. Rats that were treated with sorbinil (65 mg/kg/d) or were untreated received contusion injury with a weight drop apparatus, and sorbitol concentration in 1-cm spinal cord segments centered at the contusion epicenter was measured at 0 (uninjured) and 8 h and 4 and 7 days post-injury as described in Methods. ^†^*p* < 0.05, significant increases in sorbitol concentration attributable to contusion injury in untreated rats at 8 h and 4 and 7 days post-injury compared to laminectomized but uninjured rats (0 h; one-way analysis of variance and Duncan's test *post hoc*). ^‡^*p* < 0.01, increased sorbitol concentrations were mostly prevented by sorbinil treatment (65 mg/kg/d; two-way analysis of variance) and *p < 0.05 (Duncan's test *post hoc*; *n* = 5–7). (**B**) Hyperglucosis occurs in the spinal cord after contusion injury. Glucose was assayed in homogenates of spinal cord segments centered at the contusion epicenter as described in Methods. *p < 0.05, significant increases in glucose concentration attributable to contusion injury at 8 h and 4 days post-injury compared to laminectomized uninjured rats (0 h; one-way analysis of variance and Duncan's test *post hoc*; plasma, *n* = 3–7; spinal cord each group, *n* = 7). SE, standard error.

Measurements of sorbitol concentration were performed in spinal cord segments centered at the contusion epicenter to determine whether the polyol pathway is activated by SCI (Fig. 5A). Significant increases (78–105%) in sorbitol concentration after contusion injury were observed at 8 h and 4 and 7 days post-injury compared to laminectomized, but uninjured, rats. Increases in sorbitol concentrations were reduced to 13–30% by sorbinil treatment. These results are consistent with increased AR activity attributable to contusion injury.

The observed increases in spinal cord sorbitol concentration might be a consequence of increased tissue glucose levels caused by injury. To examine the possibility that contusion injury may increase sorbitol production by elevating substrate levels, glucose concentration was measured in spinal cord segments centered at the contusion epicenter (Fig. 5B). Contusion injury led to a substantial increase (168%) in spinal cord glucose content (i.e., hyperglucosis) within 8 h, which remained elevated (116% increase) at 4 days post-injury. There was no significant change in plasma glucose before contusion and 8 h post-contusion (Fig. 5B), indicating a switch from passive to active glucose transport, possibly involving the sodium glucose cotransporter (SGLT) or glucose transporter 1 (GLUT1) at the blood–brain barrier.^24^

## Discussion

The main findings of this study are that after spinal cord contusion injury: 1) inhibitors of AR (sorbinil, ARI-809) or SDH (CP-470711) of the polyol pathway result in similar improvements in recovery of locomotor function that are paralleled by sparing of spinal cord tissue. 2) Axonal swelling and loss of axons characteristic of Wallerian degeneration are decreased by the AR inhibitor, sorbinil. 3) Axonal swelling can be increased by hyperglycemia. 4) Axonal AR (AKR1B10) expression is increased and that increase is reduced by sorbinil treatment. 5) Activity of the polyol pathway is increased, as evidenced by increased spinal cord sorbitol concentration that, in turn, is decreased by sorbinil. 6) Tissue glucose levels are increased (evidence for each finding is below).

This study was performed to determine the extent to which the polyol pathway is activated after spinal cord contusion injury resulting in Wallerian degeneration of spinal cord axonal tracts and irreversible paralysis. Consistent with a deleterious role of increased polyol pathway activity post-injury, treatment with several inhibitors of AR and SDH resulted in partial recovery of locomotor function (Fig. 1). Improvement in locomotor function was paralleled by sparing of spinal cord tissue (Fig. 2; finding #1). This direct relationship between the sparing of spinal cord tissue and recovery of locomotor function has been found in many studies of SCI^15–17,19,23^ and is thought to be attributable to sparing of axonal tracts, which serve to coordinate lumbar pattern generators that support locomotion.

Previously, in experiments designed to implicate polyol pathway activity in the degeneration of spinal cord tissue post-injury, a model of spinal cord compression was examined in AR knockout mice.^25^ However, sparing of spinal cord tissue was not directly measured, so that the effect of deleting AR on remaining axonal tracts could not be determined. Measurements of locomotor recovery were also inconclusive given that only the acute and subacute phases, but not the chronic phase, of recovery were examined.

Axonal swelling and loss of axons characteristic of Wallerian degeneration were also decreased (Fig. 3) by the AR inhibitor, sorbinil, post-SCI (see finding #2 above). This is consistent with a mechanistic role of polyol pathway activity in Wallerian degeneration. The relative effectiveness in sparing axons was not as great (7–12%) as indicated by the measurements of overall tissue sparing of 15–31%. However, the measurements of axonal loss were performed at a relatively protected site in the ventromedial white matter. Interestingly, induction of hyperglycemia after contusion injury (Fig. 3D) resulted in increased axonal swelling (see finding #3 above). Hyperglycemia may lead to spinal cord hyperglucosis, increasing AR activity and thereby intensifying processes involving oxidative stress that promote Wallerian degeneration. However, factors other than AKR1B10 or AKR1B1 activity may be involved in this phenomenon.

Consistent with a role of AR activity in the Wallerian degeneration of spinal cord axonal tracts, immunoreactivity for AKR1B10 (Fig. 4) was elevated within axons at various stages of degeneration (see finding #4 above). AKR1B1 and AKR1B10 are coregulated and highly homologous members of the aldo-keto reductase family, although the sorbitol-forming activity of AKR1B10 is much lower than^26–28^ AKR1B1. Expression of AKR1B1 in neurons and axonal tracts has been shown to be increased by SCI.^25,29^ We now report that AKR1B10 is dramatically upregulated after SCI. AKR1B1 and AKR1B10 are both upregulated by agents that increase oxidative stress; however, AKR1B10 is several-fold more sensitive to upregulation compared to AKR1B1.^30^ Increased axonal AKR1B10, in addition to AKR1B1, expression after SCI would then further increase oxidative stress attributable to polyol pathway activity.

Measurements of sorbitol concentration were performed in spinal cord segments centered at the contusion epicenter to determine whether the polyol pathway is activated by SCI (Fig. 5A). Significant increases in sorbitol concentration attributable to contusion injury were observed compared to laminectomized but uninjured rats (see finding #5 above). Increases in sorbitol concentration were similar to levels observed in diabetic peripheral nerve^7,8^ and may be attributable to increased AR activity in neurons as well as glia. Elevations in sorbitol concentrations were largely prevented by sorbinil treatment, consistent with inhibition of increased AR activity attributable to contusion injury (see finding #5 above). The time course of increased AR activity coincided with axonal losses attributable to Wallerian degeneration.

The observed increases in spinal cord sorbitol concentration may, in part, be a consequence of increased tissue glucose levels attributable to injury. To examine the possibility that contusion injury may increase AR production of sorbitol by elevating substrate levels, glucose concentration was measured in spinal cord segments centered at the contusion epicenter (Fig. 5B). We observed that contusion injury led to a several-fold increase in spinal cord glucose content within 8 h, which remained evident at 4 days post-injury (see finding #6 above). Spinal cord glucose elevation occurs without changes in plasma glucose concentration. Therefore, it is likely that a switch from passive to active glucose transport into the spinal cord in response to ischemia is responsible, given that the ratio of spinal cord glucose to plasma levels increases from 0.6 (passive) to 1.6 (active).

**FIG. 6.**
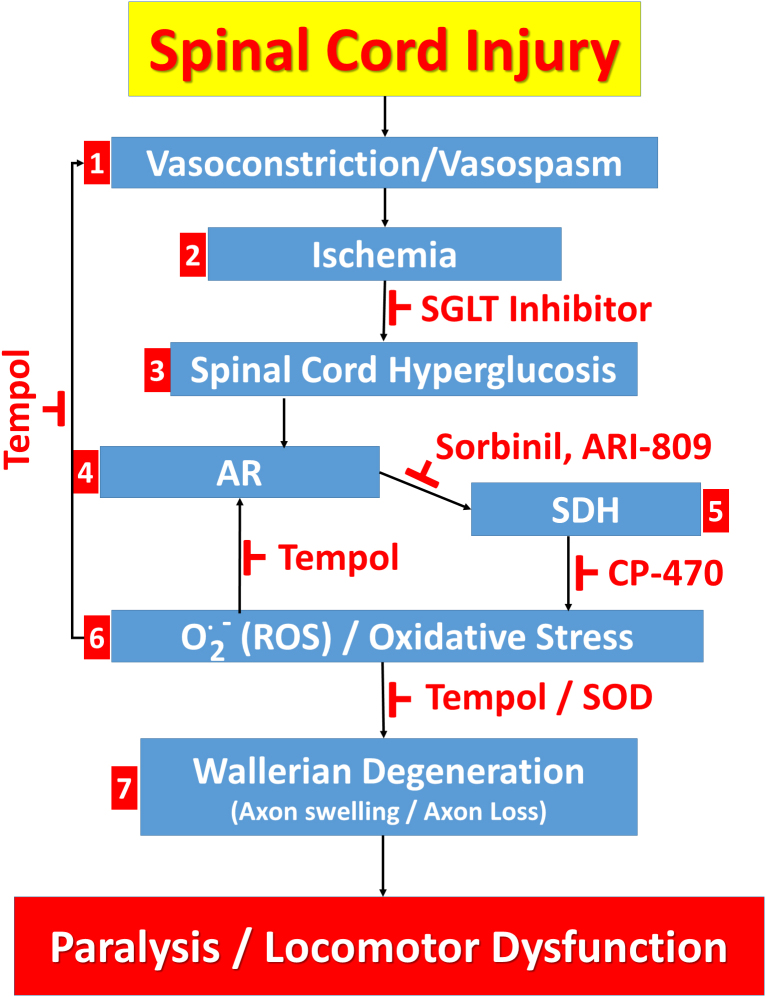
Spinal cord injury results in ischemia and spinal cord hyperglycemia, which activates the polyol pathway, causing oxidative stress, Wallerian degeneration, paralysis, and locomotor dysfunction. Contusion injury to the spinal cord causes (**1**) vasoconstriction and vasospasm in vessels supplying blood to the cord, resulting in (**2**) ischemia. Subsequently, (**3**) hyperglucosis occurs within the spinal cord, which may be attributable to greater SGLT transporter activity. Hyperglucosis, in turn, provides substrate for the polyol pathway enzymes, (**4**) AR and (**5**) SDH, generating (**6**) O^**.**^_2_^–^, ROS, and oxidative stress, in part, through NADPH oxidase activity leading to (**7**) Wallerian degeneration and associated axonal swelling, death, and loss. Paralysis and locomotor dysfunction then results, attributable to damage to ascending and descending spinal cord axonal tracts. The sequence of events leading from spinal cord injury to paralysis and locomotor dysfunction can be inhibited by inhibitors of AR (sorbinil, ARI-809) and SDH (CP-470). Additionally, scavenging of O^**.**^_2_^-^ with the SOD mimetic, tempol, can reduce vasoconstriction, oppose redox enzymatic activation of AR, and inhibit biochemical processes necessary for Wallerian degeneration. AR, aldose reductase; NADPH, β-nicotinamide adenine dinucleotide 2′-phosphate, reduced; ROS, reactive oxygen species; SDH, sorbitol dehydrogenase; SGLT, sodium glucose cotransporter; SOD, superoxide dismutase.

Figure 6 summarizes a mechanistic model of spinal cord contusion–induced injury that incorporates our current and previous observations of post-SCI response. Step 1 in Figure 6 is the rapid development of vasoconstriction/vasospasm in the region surrounding the contusion epicenter. Vasospasm alone is thought to account for an 80% loss in perfusion after SCI.^4^ Vasospasm results in spinal cord ischemia (step 2) as we have previously shown.^31^ In step 3, ischemia is known to induce luminal uptake of glucose by the sodium glucose cotransporter (SGLT) and glucose transporter 1 (GLUT1) at the blood–brain barrier.^24^ Thus, contusion-induced ischemia would be expected to trigger glucose uptake into the injured spinal cord through SGLT or GLUT1, resulting in hyperglucoses.

In steps 4 and 5, increased glucose availability results in increased throughput through AR and SDH. AR and SDH sequentially convert glucose to sorbitol and fructose in reactions (Step 6) that promote oxidative stress by amplifying the generation of ROS. AR activity consumes NADPH, which is necessary for glutathione reductase activity that regenerates reduced glutathione, the predominant cellular redox buffer that opposes oxidative stress.^5^ An important effect of increased SDH activity is generation of NADH that, in turn, leads to synthesis of the protein kinase C (PKC) activator, diacylglycerol (DAG).^32^ Elevated PKC activity is known to promote NADPH oxidase expression, which would accelerate superoxide generation.^33^ The superoxide that is generated combines readily with nitric oxide to produce peroxynitrite that can oxidize a regulatory AR cysteine, thereby increasing its catalytic activity several-fold (indicated by the arrow from step 6 to step 4).^34,35^ In addition, AR expression is regulated by the transcription factor, nuclear factor-erythroid factor 2–related factor 2,^36^ which would increase AR levels under conditions of oxidative stress, also leading to greater AR activity. Inducible nitric oxide synthase, that is upregulated post-SCI,^37^ can produce abundant amounts of nitric oxide that are sufficient, together with superoxide, to generate highly toxic levels of peroxynitrite. In step 7, finally, tissue destruction results from oxidation and subsequent degradation and denaturation of cellular proteins, lipids, and DNA.

Interestingly, the SOD mimetic, tempol, which converts superoxide into hydrogen peroxide, had very similar effects on sparing of spinal cord tissue and locomotor recovery compared to sorbinil. The effects of tempol were also non-additive with sorbinil when used in combination, consistent with involvement of similar mechanisms involving the generation of ROS. Tempol could act by depleting superoxide generated by mitochondria or through NADPH oxidase, a consequence of polyol pathway activation, which otherwise would have been available for peroxynitrite formation.

In the case of Wallerian degeneration, oxidative stress is a well-known inducer of axonal degeneration. Although the various degradative processes involved in Wallerian degeneration have only been partially elucidated, it appears that it also involves oxidative stress given that it is reported to be induced by pro-oxidants and opposed by antioxidants.^14^ Notably, AKR1B10, which was found to be expressed in degenerating axons, is known to stimulate lipogenesis and synthesis of DAG.^38^ This would provide an additional means of activating PKC and increasing NADPH oxidase expression, which would also give rise to greater generation of ROS such as peroxynitrite. NADPH oxidase activity is thought to be essential for initiating Wallerian degeneration,^14^ as well as neurodegeneration,^39^ and could have an important role in the degeneration of axonal tracts post-SCI. An important step in axonal degeneration in SCI is activation of the proteolytic enzyme, calpain, which may occur through 3-nitrotyrosinolation in the presence of peroxynitrite.^40^ α-spectrin is a structural protein of the axonal cytoskeleton that is a substrate of calpain, the loss of which would lead to axonal degeneration.^41^

The ability of hyperglycemia to exacerbate losses of axonal tracts and locomotor function after SCI^42,43^ is further evidence of the involvement of glucotoxicity in the degenerative processes. Systemic hyperglycemia, which was modeled in the experiment shown in Figure 3D, often occurs post-SCI because of either stress-induced glucose release from the liver or pre-existing diabetes. Our observation of hyperglucosis in the absence of systemic hyperglycemia (Fig. 5B), which is likely attributable to active glucose uptake, would be further increased by the presence of systemic hyperglycemia. Because there may be exacerbation of glucotoxicity under conditions of hyperglycemia, treatment with inhibitors of AR and SDH may be particularly effective in patients at higher risk for adverse sequelae associated with SCI.

Further development of effective treatment for glucotoxicity occurring in spinal cord tissue post-injury may require optimizing the blockade of polyol pathway enzyme activity, reducing levels of tissue glucose, as well as hypoxia. The AR inhibitor, sorbinil, only partially blocks the activated form of AR, and more complete inhibition has been achieved when used in combination with dimedone.^35^ Strategies for ameliorating hypoxia may necessitate the restoration of perfusion by relieving vasospasm and enhancing tissue oxygen delivery by means such as hyperbaric oxygen. Spinal cord glucose hyperglycemia post-injury might be reduced by inhibiting glucose transport into the spinal cord. Thus, a multi-pronged approach to opposing factors that cause glucotoxicity should lead to improved treatment for SCI.

## Conclusion

This study demonstrates a role of glucotoxicity, involving the polyol pathway, in the Wallerian degeneration of spinal cord axonal tracts and associated loss of locomotor function that result from spinal cord contusion injury. SCI elevates tissue glucose levels and activates AR, the rate-limiting enzyme of the polyol pathway that sequentially converts glucose to sorbitol and fructose in reactions that cause oxidative stress and axonal death. Specific inhibitors of AR and SDH of the polyol pathway result in similar sparing of spinal cord axonal tracts that support parallel improvements in locomotor function. Further development of effective treatment for glucotoxicity occurring in spinal cord tissue post-injury may require optimizing the blockade of polyol pathway enzyme activity as well as reducing levels of tissue glucose.

## Abbreviations Used

AR: aldose reductase
BBB: Basso, Beattie and Bresnahan
CNS: central nervous system
DAB: diaminobenzamine
DAG: diacylglycerol
GLUT1: glucose transporter 1
i.p.: intraperitoneal
NAD^+^: nicotinamide adenine dinucleotide
NADH: reduced NAD^+^
NADPH: β-nicotinamide adenine dinucleotide 2′-phosphate, reduced
NF-200: neurofilament 200
PKC: protein kinase C
PNS: peripheral nervous system
SDH: sorbitol dehydrogenase
SOD: superoxide dismutase
ROS: reactive oxygen species
SCI: spinal cord injury
SE: standard error
SGLT: sodium glucose cotransporter
